# Potential role of *circ_WHSC1* and *miR-145-5p* in breast cancer promotion

**DOI:** 10.1016/j.bbrep.2026.102472

**Published:** 2026-01-30

**Authors:** Maryam Abtin, Asghar Hosseinzadeh, Nahid Nafisi, Ramesh Omranipour, Leyla Sahebi, Mohsen Ahmadi, Soudeh Ghafouri-Fard, Abbas Shakoori

**Affiliations:** a Department of Medical Genetics, School of Medicine, Tehran University of Medical Sciences, Tehran, Iran; b Department of Biology Education, Farhangian University, P.O. Box 14665-889, Tehran, Iran; c Surgery Department, Rasoul Akram Hospital Clinical Research Development Center (RCRDC), Iran University of Medical Sciences, Tehran, Iran; d Breast Disease Research Center (BDRC), Tehran University of Medical Sciences, Tehran, Iran; e Department of Surgical Oncology, Cancer Institute, Tehran University of Medical Sciences, Tehran, Iran; f Maternal, Fetal and Neonatal Research Center, Family Health Research Institute, Tehran University of Medical Sciences, Tehran, Iran; g Department of Medical Genetics, Shahid Beheshti University of Medical Sciences, Tehran, Iran; h Department of Medical Genetics, Cancer Institute of Iran, Imam Khomeini Hospital Complex, Tehran University of Medical Sciences, Dr. Qarib St., Keshavarz Blvd, Tehran, Iran

**Keywords:** *circ_WHSC1*, *miR-145-5p*, Breast cancer

## Abstract

**Purpose:**

This study aims to investigate the potential interplay between *circ_WHSC1* and *miR-145-5p* in breast cancer pathogenesis using *in silico* tools, assess their clinical relevance, and evaluate the diagnostic utility of *circ_WHSC1* in clinical samples as a biomarker for breast cancer.

**Materials and methods:**

This multi-component study employed a combination of bioinformatic analyses and laboratory validation. First, *in silico* tools were used to investigate *miR-145-5p* and *circ_WHSC1* using public databases. Subsequently, their expression, correlation, and clinical relevance were experimentally assessed in a cohort of breast cancer patients.

**Results:**

*Circ_WHSC1* level was significantly higher in breast tumor compared with patient-matched adjacent normal tissues (4.17-fold change, p-value <0.01). Additionally, significant reduction of *miR-145-5p* (9.11-fold downregulation, p-value <0.01) level was detected in breast tumors compared with neighboring non-tumor tissues. A weak negative correlation was detected between levels of *circ_WHSC1* and *miR-145-5p* (r = −0.314, p-value <0.05). *Circ_WHSC1* may serve as a weak biomarker for breast cancer (AUC = 0.683; p-value <0.01) with 71 % specificity and 70 % sensitivity. Up-regulation of *circ_WHSC1* in breast tumor was linked with lymph node invasion (p-value = 0.005), HER2 negativity (p-value = 0.031) and positive family history (p-value = 0.012).

**Conclusion:**

Cumulatively, *circ_WHSC1/miR-145-5p* can be suggested as a potential molecular axis contributing to the pathogenesis of breast cancer. However, further functional assays are needed to validate this hypothesis.

## Highlights

• *Circ_WHSC1* was in breast tumor compared with patient-matched adjacent normal tissues.
• Significant reduction of *miR-145-5p* level was detected in breast tumors.
• A weak negative correlation was detected between levels of *circ_WHSC1* and *miR-145-5p.*

## 1 Introduction

Globally, breast cancer is the most common cancer and a leading cause of cancer-related death among women [1]. Despite advances in the therapeutic methods, breast cancer has a high incidence and death rate due to its multifactorial nature and tumor heterogeneity, which lead to tumor metastasis, recurrence, and treatment resistance [2]. Hence, identification of new molecular targets has pronounced implication to diminish breast cancer mortality and expand clinical efficacy [[3], [4], [5]].

miRNAs are regulatory non-coding transcripts that target certain mRNAs and regulate expression of the encoded proteins [6,7]. There are several biological processes that miRNAs participate in, such as cell proliferation, signal transduction, apoptosis, etc. So, it is obvious that dysregulation of miRNAs plays an imperative role in cancer pathogenesis [8]. *hsa-miR-145-5p* is frequently downregulated in various cancers, including breast cancer [9,10]. Established roles of this miRNA include suppression of tumor progression, induction of tumor apoptosis, and modulation of cancer stemness [[11], [12], [13]] by regulating expression of numerous genes.

CircRNAs have a key role in down regulation of *miR-145-5p* in cancer [[14], [15], [16]]. CircRNAs are a class of endogenous noncoding RNAs characterized by a covalently closed continuous loop, formed by back-splicing of single-stranded RNA transcripts. These transcripts perform their biological functions mainly by acting as microRNA (miRNA) sponge [17,18]. Several studies have shown the link between circRNA dysregulation and tumorigenesis [[19], [20], [21], [22]]. Recently, circRNAs represented a new group of biomarkers for diagnosis of malignancy, refining prognosis, and targeted therapy [23,24]. However, the functional roles of many circRNAs in cancer remain poorly understood [25].

Recent studies have shown that *circ_WHSC1* (*hsa_circ_0001387*) contributes to tumor proliferation, invasion, metastasis, and drug resistance in ovarian [26], lung [27,28], endometrial [29] and nasopharyngeal [30] cancers. It has been demonstrated that *circ_WHSC1* promotes ovarian cancer proliferation *via* targeting *miR-145-5p* [26]. Nevertheless, the potential roles of *circ_WHSC1* in breast tumorigenesis and its relationship with *miR-145-5p* in breast cancer have remained unclear. So, we designed this multi-component study and employed a combination of bioinformatic analyses and laboratory validation. We integrated *in silico* analyses of publicly available datasets (TCGA *via* CancerMIRNome and ENCORI) with experimental validation on 50 paired breast tumor and adjacent normal tissue samples. Bioinformatic analyses included differential expression, survival, diagnostic receiver operating characteristic (ROC), and functional enrichment (Gene Ontology [GO]/KEGG) assessments for *miR-145-5p*, as well as interaction network mapping for *circ_WHSC1*. Clinically, *circ_WHSC1* and *miR-145-5p* expression levels were quantified *via* qRT-PCR, and their diagnostic potential and correlation with clinicopathological features were statistically evaluated.

## 2 Methods and materials

### 2.1 Bioinformatics analysis on *hsa-miR-145-5p* expression level and overall survival outcomes of breast cancer in TCGA cohort

We analyzed the expression profile of *hsa-miR-145-5p* in breast cancer using the CancerMIRNome platform. This platform serves as a resource for assessment of miRNAs expression, and their diagnostic and prognostic relevance across various cancer types [31]. We utilized the related data obtained from TCGA cohort which included the expression data of 1078 breast cancer samples and 104 normal controls. We also assessed the diagnostic relevance of *hsa-miR-145-5p* in breast cancer using this tool. Additionally, we investigated the relationship between the expression levels of *hsa-miR-145-5p* and the overall survival (OS) outcomes of breast cancer, utilizing data from the CancerMIRNome database. A log-rank p-value <0.05 was considered statistically significant.

### 2.2 Functional enrichment analysis

To elucidate the functional enrichment of mentioned miRNA, which encompasses GO and KEGG pathway, we employed the pathway module available in the ENCORI database (https://rnasysu.com/encori/index.php). This database performs analyses based on the predicted target genes of the miRNA. The GO analysis was categorized into Biological Process (BP), Molecular Functions (MF), and Cellular Components (CC). The identified terms were organized based on log_10_ adjusted p-value (FDR) threshold of less than 0.01.

### 2.3 Collection and handling of breast tumor and non-tumor tissue samples

All specimens were obtained from the patients referred to two surgery centers, in Tehran, Iran. All samples were gathered in RNA Later reagent (Biobasic, Canada) and kept based on instructions. A total of 50 pairs of tumor and corresponding non-tumor tissue samples were examined by a pathologist. Tissues were fresh-frozen and transferred to −70 °C freezer until being used for RNA extraction. Patients experienced neither radiotherapy nor chemotherapy before sampling. The protocol was certified by the ethical review committee of the Tehran University of Medical Sciences under the ethical ID of IR.TUMS.MEDICINE.REC.1401.272, in accordance with the Helsinki Declaration. Patient agreement was attained before the samples were taken.

### 2.4 RNA extraction and cDNA synthesis

Total RNA was retrieved using TRIzol reagent (Invitrogen). RNA integrity and concentration were assessed using agarose gel electrophoresis and spectrophotometry, respectively. For cDNA production, the ExcelRT™ 1st Strand cDNA Synthesis Kit (SMOBIO) was applied. The specific stem-loop RT primers for *miR-145-5p* and *snRNA U6* were added to the reaction mix and cDNA was synthesized based on kit direction. The sequence of *circ_WHSC1* was attained from the Circbase database, and specific divergent primers were designed by CircPrimer 2.0 software.

### 2.5 Real-time PCR

Real-time PCR was performed using RealQ Plus 2x Master Mix Green low Rox (Ampliqon) *via* Light-Cycler96 Roche. Reactions were carried out in 10 μl volume in duplicate. Gene expressions were quantified using the 2^−ΔΔCT^ method, normalized to *GAPDH* (for *circ_WHSC1*) or *U6* (for *miR-145-5p*). PCR products were evaluated by melting curve examination and electrophoresis on agarose gel. Back-splicing-derived circularization of circ_WHSC1 was confirmed through Sanger sequencing using divergent primers spanning the junction site (primer sequences provided in Table 1).

**Table 1:** Primers sequences.

Gene symbol	Primer name	Sequence (5′ to 3′)	Tm	Size of amplicon (bp)	Source of primer
*CircWHSC1*	Forward	CACAGTCTTCGGAAGTGTTCT	57.90	134	This study
	Reverse	ACTGCCGAGGATTTCTGGTG	60.04		
*miR-145-5p*	Stem- Loop RT primer	GTCGTATCCAGTGCAGGGTCCGAGGTATTCGCACTGGATACGACAGGGAT			Our previous study [32]
	Forward	GGCTTAGTCCAGTTTTCCCAG	58.86	69	
	Reverse	GTGCAGGGTCCGAGGT	58.01		
*U6snRNA*	Stem- Loop RT primer	GTCGTATCCAGTGCAGGGTCCGAGGTATTCGCACTGGATACGACAAAAATAT			Our previous study [32]
	Forward	GCTTCGGCAGCACATATACTAAAAT	60.05	91	
	Reverse	CGCTTCACGAATTTGCGTGTCAT	63.00		
*GAPDH*	Forward	AAGGCTGAGAACGGGAAGCT	61.48	91	Our previous study [32]
	Reverse	CAGCATCGCCCCACTTGATT	61.03		

### 2.6 Statistical analysis

Statistical assessments were done by GraphPad Prism 8.4.3 and SPSS v.26. Relative expression of *circ_WHSC1* and *miR-145-5p* was compared in breast tumors and adjacent tissues by applying the paired sample *t*-test. The correlation of expression of *circ_WHSC1* and *miR-145-5p* was determined using the Spearman correlation factor. The relationship between the expression of *circ_WHSC1* and clinicopathological characteristics of patients was judged using Mann-Whitney and Kruskal-Wallis tests. Furthermore, ROC curves were generated using GraphPad Prism. The p-value <0.05 was considered to describe the statistical significance in all assessments.

## 3 Results

### 3.1 Bioinformatics analysis on *hsa-miR-145-5p* expression level and overall survival outcomes of breast cancer in TCGA cohort

**Fig. 1:**
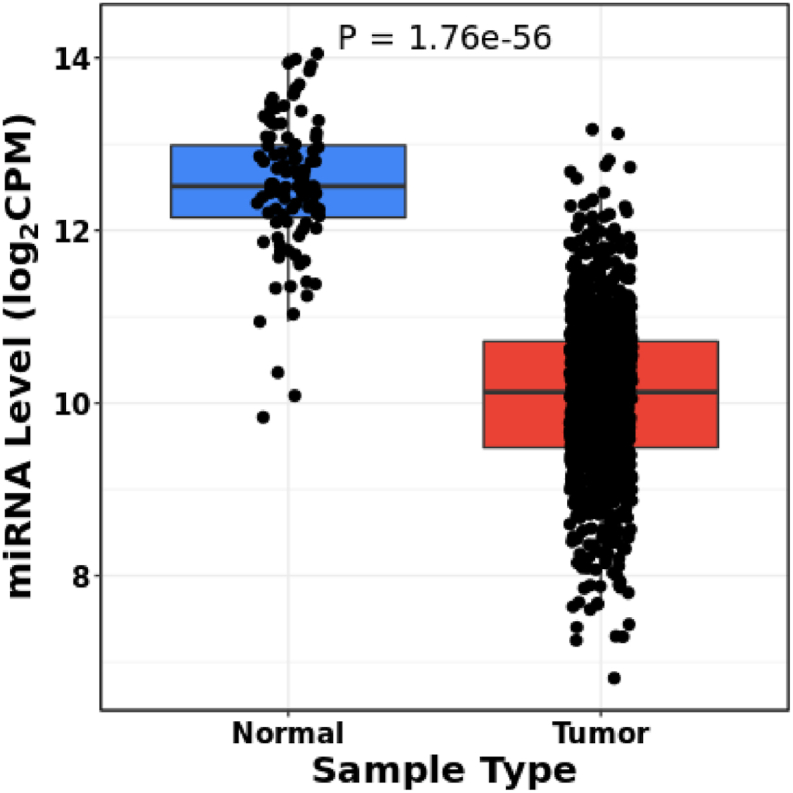
An examination of levels of *hsa-miR-145-5p* in breast cancer tissues (n = 1078) compared to normal tissues (n = 104) was conducted utilizing the CancerMIRNome database. A p-value of less than 0.05 was deemed statistically significant.

Expression analysis utilizing the CancerMIRNome database showed that *hsa-miR-145-5p* was significantly down-regulated in 1078 breast cancer specimens compared to 104 controls (Fig. 1; p-value = 1.7e-56).

**Fig. 2:**
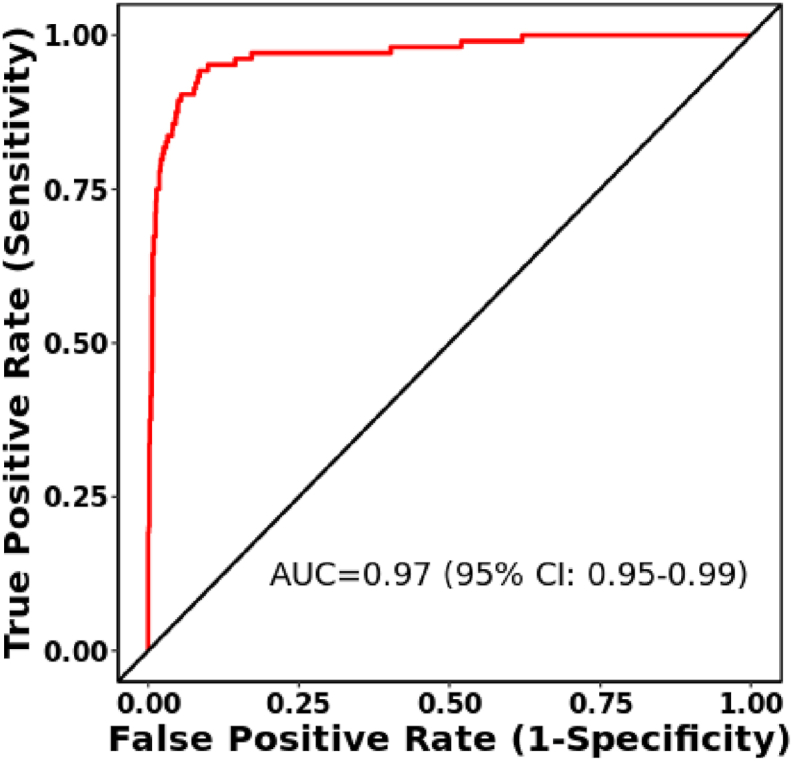
The diagnostic value of the *hsa-miR-145-5p* in breast cancer using receiver operating characteristic (ROC) curve analyses (Tumor (n = 1078) *vs*. Normal (n = 104)) *via* the CancerMIRNome database. A p-value <0.05 was regarded as significant (AUC: area under curve).

The analysis of the ROC curve using the CancerMIRNome database indicated that *hsa-miR-145-5p* may possess significant diagnostic value for individuals diagnosed with breast cancer, as evidenced by an Area Under the Curve (AUC) of 0.97, a p-value <0.0001, and a 95 % Confidence Interval ranging from 0.95 to 0.99 (Fig. 2).

### 3.2 Overall survival (OS) analysis of *hsa-miR-145-5p* in breast cancer using *in silico* tools

**Fig. 3:**
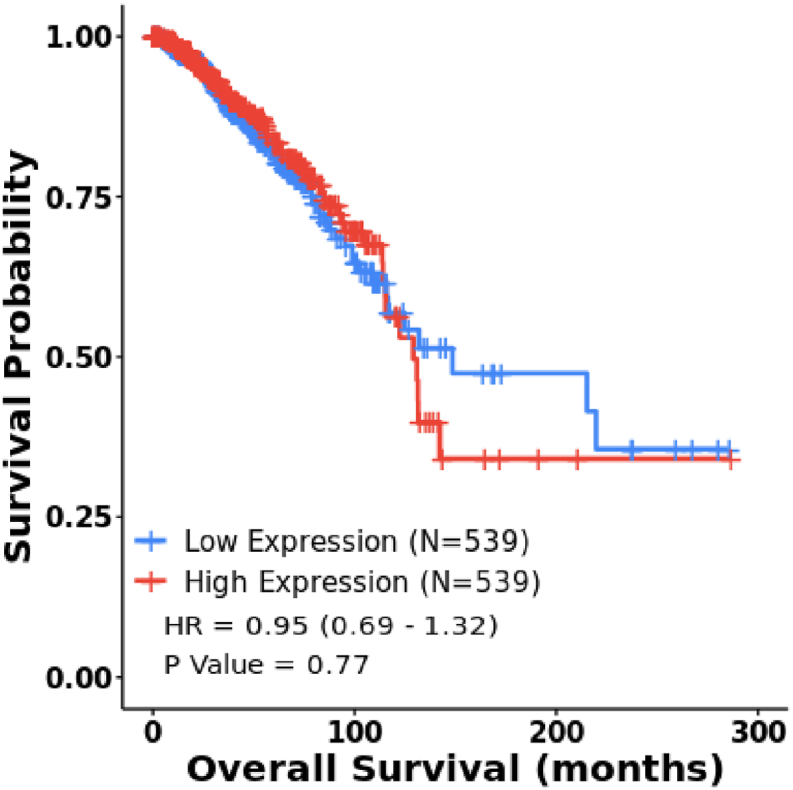
The overall survival curve of patients with breast cancer based on *hsa-miR-145-5p* expression according to the CancerMIRNome (total number of patients = 1078). Log-Rank p-value <0.05 was considered statistically significant. N: Sample count.

As demonstrated in Fig. 3, the survival analysis by the CancerMIRNome database indicated no significant associations between lower expression of *hsa-miR-145-5p* and OS of breast cancer patients.

### 3.3 GO and KEGG pathway analyses based on *in silico* tools

**Fig. 4:**
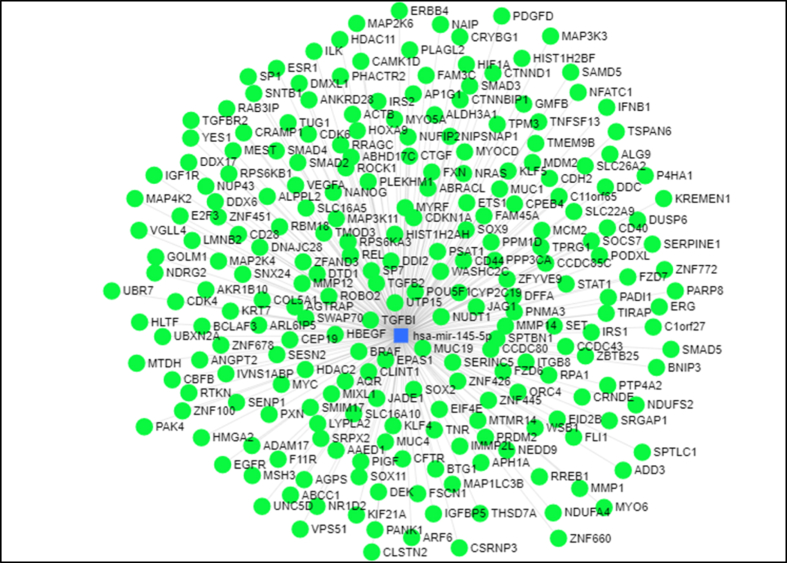
Interacting network between *hsa-miR-145-5p* and its hypothetical targets. The blue square represents hsa-miR-145–5p and green circles indicate target genes of this miRNA (www.mirnet.ca).

First, we demonstrated the interactive network between *miR-145-5p* and its targets interacting network in breast cancer (Fig. 4).

The GO analysis unearthed that the targets of *hsa-miR-145-5p* were mainly enriched in Cellular Macromolecule Localization, Establishment Of Protein Localization, Positive Regulation Of Nucleobase Containing Compound Metabolic Process in BP (Table 2); in Enzyme Binding, Protein Containing Complex Binding, Ribonucleotide Binding in MF (Table 3); and in Organelle Subcompartment, Golgi Apparatus, Endoplasmic Reticulumin CC (Table 4). The KEGG pathway analyses also indicated that these genes were enriched in Endocytosis, Focal Adhesion, Regulation Of Actin Cytoskeleton pathways (Table 5).

**Table 2:** Biological Processes (BP) terms for *hsa-miR-145-5p*.

BP terms	log_10_ (p-value)	log_10_ (FDR)
Cellular Macromolecule Localization	−99.36547	−95.60543
Establishment Of Protein Localization	−84.14343	−80.8605
Positive Regulation Of Nucleobase Containing Compound Metabolic Process	−84.14396	−80.68494
Positive Regulation Of Biosynthetic Process	−80.58294	−77.42496
Intracellular Transport	−80.376	−77.31493
Regulation Of Protein Modification Process	−79.54705	−76.56516
Regulation Of Intracellular Signal Transduction	−76.46086	−73.54591
Positive Regulation Of Protein Metabolic Process	−71.38218	−68.52523
Positive Regulation Of Molecular Function	−71.18779	−68.38199
Regulation Of Phosphorus Metabolic Process	−70.83962	−68.07957

**Table 3:** Molecular Function (MF) terms for *hsa-miR-145-5p*.

MF terms	log_10_ (p-value)	log_10_ (FDR)
Enzyme Binding	−104.25187	−101.19496
Protein Containing Complex Binding	−70.88172	−68.12584
Ribonucleotide Binding	−67.29546	−64.71567
Transcription Regulator Activity	−61.19369	−58.73885
Identical Protein Binding	−59.96512	−57.60718
Adenyl Nucleotide Binding	−57.65983	−55.38108
Sequence Specific Dna Binding	−57.1824	−54.9706
Rna Binding	−56.74355	−54.58974
Cytoskeletal Protein Binding	−52.24391	−50.14125
Dna Binding Transcription Factor Activity	−46.43723	−44.38033

**Table 4:** Cellular Component (CC) terms for *hsa-miR-145-5p*.

CC terms	log_10_ (p-value)	log_10_ (FDR)
Organelle Subcompartment	−69.32104	−66.45475
Golgi Apparatus	−67.84869	−65.28343
Endoplasmic Reticulum	−60.08455	−57.69538
Endosome	−50.38498	−48.12075
Neuron Projection	−46.2645	−44.09718
Vacuole	−44.29713	−42.27594
Anchoring Junction	−44.30292	−42.21479
Chromosome	−44.06045	−42.09725
Plasma Membrane Region	−42.76317	−40.85113
Vesicle Membrane	−40.68108	−38.81479

**Table 5:** KEGG terms for *hsa-miR-145-5p*.

KEGG terms	log_10_ (p-value)	log_10_ (FDR)
Endocytosis	−21.82506	−19.62916
Focal Adhesion	−19.40604	−17.51117
Regulation Of Actin Cytoskeleton	−18.41674	−16.69797
Mapk Signaling Pathway	−17.72381	−16.12997
Pathways In Cancer	−17.13598	−15.63905
Axon Guidance	−14.10341	−12.68566
Insulin Signaling Pathway	−11.63633	−10.28553
Neurotrophin Signaling Pathway	−11.46096	−10.16815
P53 Signaling Pathway	−11.27545	−10.03379
Lysosome	−9.97121	−8.77531

### 3.4 Expression analysis of *circ_WHSC1* and *miR-145-5p* in clinical specimens

**Fig. 5:**
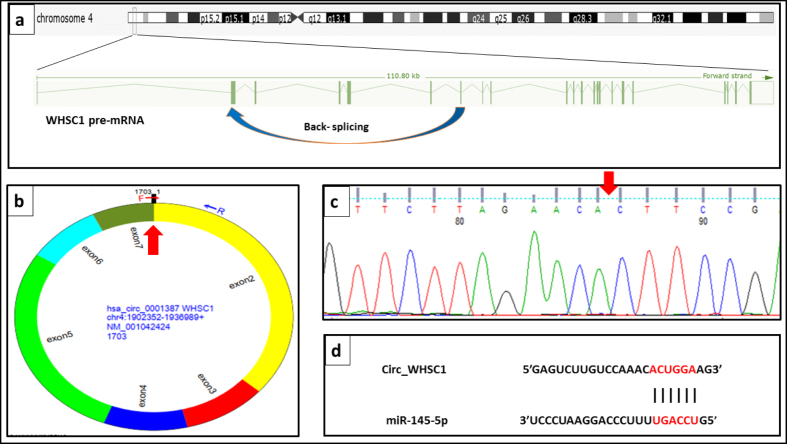
a. schematic representation of *circ_WHSC1* biogenesis b. *circ_WHSC1* divergent primer illustrated by circprimer 2.0. c. the back-splice junction site of *circ_WHSC1* was verified by Sanger sequencing d. Interaction site of *circ_WHSC1* and *miR-145-5p* (circinteractome.nia.nih.gov).

Sanger sequencing was employed for confirming the back splice junction resulted from joining of exon 2 and exon 7 of *WHSC2* gene as host gene of *circ_WHSC1*. Fig. 5 shows a schematic representation of *circ_WHSC1* biogenesis (Fig. 5a), divergent primer designed for amplification of *circ_WHSC1* (Fig. 5b), verification of the back-splice junction site of *circ_WHSC1* (Fig. 5c), and interaction site of *circ_WHSC1* and *miR-145-5p* (Fig. 5d).

**Fig. 6:**
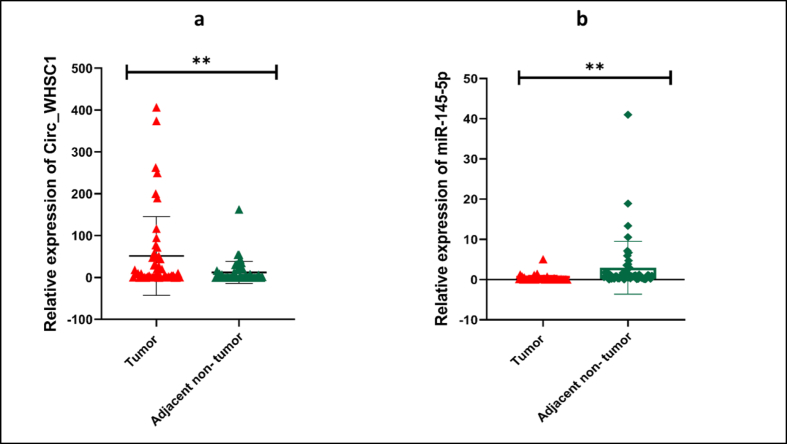
Scatter plots with bar showing relative expression of *circ_WHSC1* (a) and *miR-145-5p* (b) in breast cancer tissues (n = 50) relative to normal neighboring tissue (n = 50) (Mean and standard deviation values are shown, ∗∗: p-value <0.01).

Expression levels of *circ_WHSC1* and *miR-145-5p* in clinical samples were normalized to *GAPDH* and *U6*, respectively. We found significant up-regulation of *circ_WHSC1* in breast tumors compared with corresponding neighboring normal tissues (4.17-fold change, P = 0.0026) (Fig. 6a). Additionally, significant reduction of *miR-145-5p* (9.11-fold downregulation, P = 0.0068) level was detected in breast tumors compared with non-tumor tissues (Fig. 6b). Detailed statistics of expression ratios are shown in Table 6.

**Table 6:** Detailed statistics of expression ratio of *circ_WHSC1* and *miR-145-5p* in breast cancer *vs*. corresponding neighboring normal tissues (Paired *t*-test results are shown).

Parameters	*circ_WHSC1*	*miR-145-5p*
P value	0.0026	0.0068
One- or two-tailed P value?	Two-tailed	Two-tailed
t, df	t = 3.178, df = 49	t = 2.825, df = 49
Number of pairs	50	50
R squared (partial eta squared)	0.1709	0.1400
Average expression of tumor samples group (T)	51.46807412	0.321544
Average expression of non-tumor samples group (N)	12.32131884	2.93108
Fold change	4.1771 (T/N)	9.1156 (N/T)

### 3.5 Correlations between expressions of *circ_WHSC1* and *miR-145-5p*

**Fig. 7:**
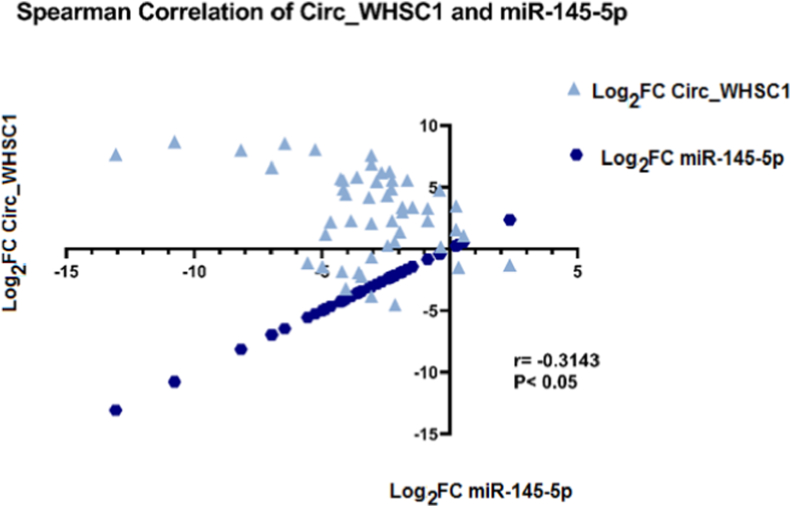
Point graph in XY axes showing Spearman correlation of *miR-145-5p* and *circ_WHSC1* expression (Number of tumor samples = 50, r = −0.3143, p-value <0.05).

Correlation analyses between expression quantities of *circ_WHSC1* and *miR-145-5p* were achieved by Spearman correlation coefficient. As demonstrated in Fig. 7, a weak negative correlation was perceived between levels of *circ_WHSC1* and *miR-145-5p* (r = −0.3143, 95 % CI = −0.5509 to −0.03094 and p-value = 0.0262).

### 3.6 The implication of *circ_WHSC1* as a marker

**Fig. 8:**
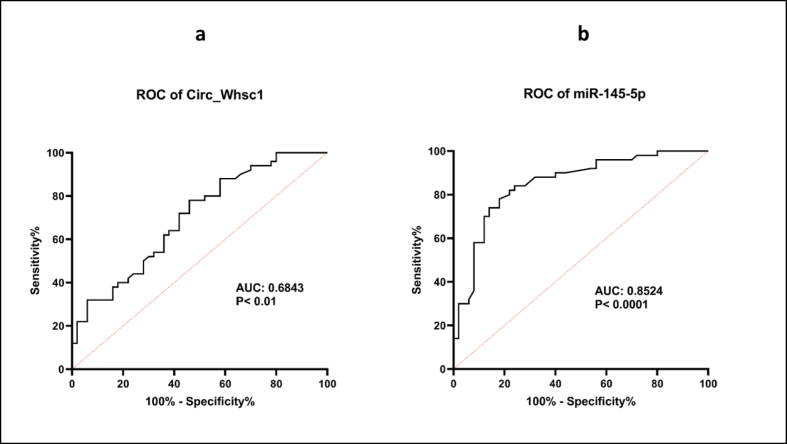
Receiver operating characteristic (ROC) curve of a. *circ_WHSC1* (*P* < 0.01) and b. *miR-145-5p* (*P* < 0.0001) in breast cancer patients (Tumor (n = 50) *vs*. Normal (n = 50), AUC: area under curve).

ROC curve was plotted to appraise the diagnostic biomarker power of mentioned genes. We used gene expression values in tumors and adjacent non-tumor tissues for producing ROC curves. This assessment discovered that *circ_WHSC1* has a weak power to distinguish between tumor tissue and adjacent normal tissues (AUC = 0.683, Standard error = 0.05301, 95 % CI = 0.5795 to 0.7873, p-value = 0.0016) with 71 % specificity and 70 % sensitivity (Fig. 8a). Moreover, ROC curve was generated for *miR-145-5p* and demonstrated that *miR-145-5p* has moderate power in discriminating between these two sets of samples (AUC = 0.8524, Standard error = 0.0386, 95 % CI = 0.7767 to 0.9281, 88 % specificity and 88 % sensitivity, p-value <0.0001) (Fig. 8b).

### 3.7 Correlations of *circ_WHSC1* f levels and clinicopathological characteristics

The clinical information of patients such as age, tumor dimension, grade, hormone receptors and TNM stage exhibited in Table 7. Correlation examinations indicated that up-regulation of *circ_WHSC1* in breast tumors was linked with lymph node invasion (p-value = 0.005), *HER2* negativity (p-value = 0.031) and positive family history (p-value = 0.012).

**Table 7:** Correlation of *circWHSC1* expression and clinicopathological data (N = 50).

	Subclass	Number of patients (%)	Median of *Circ_Whsc1* level	Interquartile Range (IQR)	Mean of *Circ_Whsc1* level	SD	p-value
Age	≤45	15	4.4	5.57	3.04	3.50	0.84
	>45	35	2.97	5.4	2.85	3.55	
Tumor diameter	<2 cm	7	0.57	6.99	2.02	3.49	0.1
	2–5 cm	38	3.42	4.88	3.47	3.3	
	>5 cm	5	0.27	6.53	−0.16	3.42	
Invasion to lymph	yes	29	4.87	4.50	4.10	3.41	**0.005**
	no	21	1.37	5.10	1.25	2.98	
Grade	1	13	3.47	6.95	3.55	3.93	0.63
	2	31	2.97	5.40	2.58	3.44	
	3	6	3.32	5.22	3.17	3.23	
Stage	1	11	0.57	6.8	2.29	3.58	0.33
	2	32	3.32	4.15	3.16	3.37	
	3	6	4.99	6.2	3.81	3.56	
	4	1					
*ER*	P	41	3.37	4.89	3.16	3.4	0.18
	N	9	1.16	6.82	1.26	3.9	
*PR*	P	37	3.37	5.2	2.96	3.44	0.7
	N	12	2.66	7.08	2.51	3.89	
*HER2*	P	7	−0.68	3.77	0.11	2.99	**0.031**
	N	43	3.47	4.77	3.36	3.40	
*KI67*	<16	19	3.47	7.55	3.31	3.88	0.47
	16 or >16	31	3.27	5.20	2.66	3.3	
Family history	present	13	5.57	4.88	4.99	3.00	**0.012**
	absent	37	2.27	5.87	2.17	3.40	

## 4 Discussion

Breast cancer is the most common malignancy among women [1]. This malignancy is associated with dysregulation of several circRNAs [33]. Most of these molecules exert their role in breast cancer progression through sponging miRNAs [33]. Thus, exploring the role of circRNAs/miRNAs axes in this cancer is important in identification of biomarkers.

*Circ_WHSC1* is originated from exonic back-splicing of *WHSC1* gene, a histone methyl transferase that is controlled by *EZH2* and has imperative functions in ovarian carcinogenesis [34]. The impact of *circ_WHSC1* in the carcinogenesis has been evaluated in several tissues [35]. Moreover, *circ_WHSC1* has been found to stimulate ovarian tumorigenesis through adsorbing *miR-145* and another miRNA, namely *miR-1182* [26]. We evaluated expression level of this circRNA and *miR-145* in breast cancer samples. Similar to what reported in ovarian cancer, we found significant up-regulation of *circ_WHSC1* and down-regulation of *miR-145-5p* in breast tumors compared with corresponding adjacent normal tissues. A weak negative correlation was detected between levels of *circ_WHSC1* and *miR-145-5p*, suggesting the similar effect of this circRNA on expression of mentioned miRNA in breast and ovarian malignancies. An earlier study has shown that *circ_WHSC1* accelerates malignancy and glycolysis of triple negative breast cancer cells through regulation of *miR-212-5p/AKT3* axis [36]. Another study has revealed the importance of *circ_WHSC1/miR-195-5p* in the regulation of *FASN/AMPK/mTOR* axis and promotion of breast carcinogenesis [37]. Thus, the oncogenic effects of *circ_WHSC1* in breast cancer are exerted through different routes. We also assessed TCGA data of *miR-145-5p* in breast cancer and demonstrated similar results with the findings of current study. However, this data did not show correlation between expression level of *miR-145-5p* and OS of patients. The lack of correlation with patient survival in TCGA could be due to molecular subtype distribution or sample size. Thus, further assessments in larger cohorts of patients are needed.

Based on the *in silico* assays, this miRNA was found to be linked with a number of pathways, including those related with endocytosis, focal adhesion, regulation of actin cytoskeleton, as well as MAPK and P53 signaling pathways. Meanwhile, *in silico* analyses suggested interaction between *circ_WHSC1* and a number of RNA binding proteins that are involved in the carcinogenesis. Among these proteins is HUR, a protein that is involved in mRNA transport from nuclear compartment to cytoplasm, and regulation of stability and translation of mRNAs. Dysregulation of HUR has been found to be associated with cancer [38]. AGO2 protein has also been reported to participate in transcriptional activation in breast cancer cells, and stimulate cell growth through induction of expression of the progesterone receptor [39]. Thus, the *circ_WHSC1/miR-145-5p* axis can be involved in breast carcinogenesis through different mechanisms. However, it is worth emphasizing that these are computational predictions and not experimentally confirmed. Such interactions should be validated through immunoprecipitation (RIP) or crosslinking immunoprecipitation (CLIP).

ROC curve analysis revealed weak performance of *circ_WHSC1* as a biomarker for breast cancer. However, the data provided by CancerMIRNome database revealed excellent performance of *miR-145-5p* as a diagnostic marker in breast cancer. Thus, while *miR-145-5p* has stronger diagnostic performance, *circ_WHSC1* has potential use as part of a biomarker panel, rather than as a standalone marker.

Up-regulation of *circ_WHSC1* in breast tumors was linked with lymph node invasion, *HER2* negativity and positive family history. Since *circ_WHSC1* enhances cell viability, migratory potential and invasion in ovarian tumors [26], it is not surprising that its up-regulation in breast cancer is associated with lymph node metastasis. Moreover, studies in breast cancer specimens and animal models support similar roles for *circ_WHSC1* in this type of cancer [36,37]. Finally, this finding is supported by the involvement of *circ_WHSC1* in several cancer-related pathways, its interaction with RNA binding proteins and its transcriptional activity role. Overall, our findings suggest that the *circ_WHSC1*/*miR-145-5p* axis is a potential molecular pathway contributing to breast carcinogenesis and may serve as both a diagnostic biomarker and a therapeutic target, pending functional and clinical validation.

To confirm the regulatory relationship between *circ_WHSC1* and *miR-145-5p*, functional assays such as *circ_WHSC1* knockdown or overexpression experiments followed by *miR-145-5p* quantification are recommended. Luciferase reporter assays using the predicted binding sites would help validate direct interactions. Besides, to definitively validate the circular nature of *circ_WHSC1*, future experiments should include RNase R digestion assays. This treatment selectively degrades linear RNAs while preserving circular transcripts, providing stronger evidence of circularity beyond divergent primer PCR and Sanger sequencing. Additionally, elucidation of downstream targets requires further experimental steps. In fact, although we observed a significant downregulation of *miR-145-5p*, the downstream mRNA targets of this miRNA in breast cancer require further exploration. Transcriptomic profiling following *miR-145-5p* restoration could identify key downstream genes involved in tumor progression.

The interaction of *circ_WHSC1* with RNA-binding proteins such as AGO2, HUR, FMRP, and FUS, as predicted *in silico*, suggests a broader role in post-transcriptional regulation. RNA immunoprecipitation (RIP) or crosslinking immunoprecipitation (CLIP) assays could confirm these interactions experimentally. Future studies using *in vivo* models, such as xenograft or genetically engineered mouse models, would be valuable in confirming the oncogenic role of *circ_WHSC1* and its impact on tumor growth, metastasis, and therapeutic response.

Although we demonstrated associations between *circ_WHSC1*/*miR-145-5p* levels and some clinicopathological features, larger multicenter studies are necessary to validate these biomarkers across diverse patient populations and breast cancer subtypes. Finally, given the oncogenic features of *circ_WHSC1* and tumor-suppressive role of *miR-145-5p*, therapeutic strategies such as antisense oligonucleotides (ASOs) against *circ_WHSC1* or miRNA mimics for *miR-145-5p* restoration may be promising and warrant preclinical investigation.

In fact, the regulatory effect of *circ_WHSC1* on *miR-145-5p* requires experimental support *via* knockdown/overexpression and luciferase assays to demonstrate direct interaction and functional consequence. This data might facilitate design of novel therapeutics that targets this axis. Moreover, a biomarker panel consisted of *circ_WHSC1* on *miR-145-5p* can be designed for diagnostic purposes in breast cancer. Finally, although circRNAs and miRNAs hold significant potential in the context of personalized cancer therapy due to their expression-based specificity, the patient cohort selected in this study presents demographic heterogeneity. Given that expression data in epigenetic research are highly sensitive and influenced by environmental factors, we state this point as another limitation of our study. While we used patient-matched adjacent normal breast tissue as the control—a standard practice that rigorously controls for inter-individual genetic and environmental variability—it is recognized that such tissue may not represent a truly “healthy” state. Adjacent normal tissue can be influenced by the local tumor microenvironment or exhibit a “field effect,” potentially harboring subtle molecular alterations. Although the use of tissue from completely healthy donors could theoretically circumvent this, obtaining such samples is ethically and practically challenging, as it requires invasive procedures without clinical benefit for the donor. Therefore, our internal control design, while optimal for identifying tumor-specific dysregulation, may slightly underestimate the magnitude of differential expression between cancerous and completely normal breast epithelium. Future studies incorporating larger, multi-center cohorts and, where feasible, non-diseased reference tissues (e.g., from reduction mammoplasties with confirmed pathological normality) would help to further contextualize these findings.

## 5 Conclusions

This study suggests the evidence of *circ_WHSC1*/*miR-145-5p* interaction in breast cancer. This axis is a potential molecular axis contributing to the pathogenesis of breast cancer. Additional research is needed to validate the functional role of this axis in breast cancer and design novel therapeutic options that target this axis.

## Ethics approval and consent to participant

All procedures were in accordance with the ethical standards of the institutional research committee and with the 1964 Helsinki declaration and its later amendments. Informed consent forms were obtained from all study participants. The study protocol was approved by the ethical committee of Tehran University of Medical Sciences (IR.TUMS.MEDICINE.REC.1401.272).

## Consent of publication

Not applicable.

## Availability of data and materials

All data generated or analyzed during this study are included in this published article.

## Authors’ contributions

AS was the supervisor of the study. AH, NN, and RO provided the clinical samples and related information. LS and MAh analyzed the data. MAb conducted the experiments. SG-F wrote the manuscript. All authors approved the final draft.

## Funding

This study was supported by 10.13039/501100004484Tehran University of Medical Sciences.

## Declaration of competing interest

Authors declare no conflict of interests.

## Data Availability

No data was used for the research described in the article.
